# Surface-Enhanced Raman Spectroscopy Chips Based on Silver Coated Gold Nanostars

**DOI:** 10.3390/nano12203609

**Published:** 2022-10-14

**Authors:** Miriam Parmigiani, Benedetta Albini, Giovanni Pellegrini, Marco Genovesi, Lorenzo De Vita, Piersandro Pallavicini, Giacomo Dacarro, Pietro Galinetto, Angelo Taglietti

**Affiliations:** 1 Department of Chemistry, University of Pavia, Viale Taramelli 12, 27100 Pavia, Italy; 2 Department of Physics, University of Pavia, Via Bassi 6, 27100 Pavia, Italy

**Keywords:** SERS, gold nanostars, seed-growth synthesis, pollutants, thiram, norfloxacin, silver shell

## Abstract

Surface-enhanced Raman scattering (SERS) is becoming widely used as an analytical tool, and the search for stable and highly responsive SERS substrates able to give ultralow detection of pollutants is a current challenge. In this paper we boosted the SERS response of Gold nanostars (GNS) demonstrating that their coating with a layer of silver having a proper thickness produces a 7-fold increase in SERS signals. Glass supported monolayers of these GNS@Ag were then prepared using simple alcoxyliane chemistry, yielding efficient and reproducible SERS chips, which were tested for the detection of molecules representative of different classes of pollutants. Among them, norfloxacin was detected down to 3 ppb, which is one of the lowest limits of detection obtained with this technique for the analyte.

## 1. Introduction

In Surface Enhanced Raman Spectroscopy (SERS) the excitation of Localized Surface Plasmonic Resonances (LSPR) supported by nanostructured metals leads to an intensification of the Raman light scattered by molecules adsorbed or located near the metal surface [1]. This effect has led to the development of an ultra-sensitive technique, in which the main features of Raman spectroscopy (flexibility, speed and specificity in the identification of molecules through the vibrational fingerprint) are coupled to an increase in sensitivity which can reach the single molecule level. Therefore, SERS is considered nowadays as one of the most promising tools for the detection of trace molecules and ions. In particular, it is gaining considerable attention for chemical sensing and environmental monitoring, especially as a sensing probe for the detection of the so-called emerging pollutants, which are chemicals that can cause detrimental effects on the environment and/or human health. These chemicals can be found in pharmaceuticals, pesticides, personal care products, industrial additives, solvents, artificial sweeteners, industrial and household products [2,3]. Common identification techniques of these kinds of pollutants mainly rely on expensive instrumentation, such as gas chromatography-mass spectrometry (GC-MS) or high-performance liquid chromatography (HPLC). Although these techniques hold an acceptable reliability, their use implies only off-site analyses, thus leaking the advantage of in situ, instantaneous and remote identification and monitoring. Therefore, with the increase in environmental pollution, especially in water and in the atmosphere, technologies that allow in situ monitoring as well as rapid identification [4] are highly desired and SERS is a valid candidate.

Norfloxacin is a fluoroquinolone (FQN) representative of a class of antibiotics that is known for broad-spectrum activity against several infections and their use is getting larger in several ambits. In particular, norfloxacin is one of the most used antibiotics; for example, it is the second most used in Latin America [5]. During the last decade several researchers have revealed its presence in surface waters (lakes, rivers, sea) and in wastewater [6]. This is the result of several factors: an excess of use in agriculture and farming, the discharge of wastewater from pharmaceutical industries, and also some presence from household water. Moreover, a non-negligible aspect is that FQN are quite stable and poorly biodegradable, being characterized by halving times of 10.6 days in surface water and up to 580 days in soils [7]. The European Union has set the maximum residue limit to 3.01 × 10^−7^ M. As a response to norfloxacin diffusion in the environment, several investigations have been reported on its rapid identification [6]. Although SERS spectroscopy is a natural candidate for this type of investigation, only a few examples of its application to norfloxacin detection are reported in literature. In a recent work, the detection limit was set around 10 ppb for a SERS detection based on superlattices formed with gold nanorods followed by solvent evaporation on a slippery surface [8]. Two other research groups recently declare even lower detection limits by using as sensing devices an optofluidic system with in-fibre integrated Ag nanoparticles [9] and a free-standing membrane liquid-state platform [10]. Nevertheless, there is an urgent need for alternative and simpler sensors enabling fast and in situ detection.

Thiram is a dithiocarbamate derived molecule quite diffused as pesticide and fungicide in agriculture, well known to be a water emerging pollutant as well as a potential threat for human health [11,12,13]. Toxicity of thiram in surface waters for various animal species (from fishes to amphibia) is well documented [12,13,14]. As it possesses sulphur functions ready to interact with noble metal surfaces, thiram detection by SERS has been reported in several examples during the last decade [15] regarding its recognition both on fruit peels and in water. In the United States and the European Union, the limit range of thiram is 0.1–15 mg/kg in fruits and vegetables, but there is no specific limit for grains. The maximum residue limit (MRL) for wheat is 1 mg/kg (calculated as carbon disulfide, CS2) in the National Food Safety Standard (GB 2763-2019) [16].

Toluene is an aromatic hydrocarbon with well-known toxic effects on human health. Detection of aromatic hydrocarbons with SERS technique is not a trivial task, as these kinds of molecules do not express any affinity for noble metal surfaces [4]. One of the possible ways to overcome this problem is the use of a thin film of PDMS to coat the SERS sensing devices, in order to exploit PDMS ability to absorb hydrophobic molecules from aqueous mixtures [4].

As the metallic substrate plays an important role in achieving high sensitivity through the field enhancement, enormous efforts are under way to select noble metal nanoplatforms able to maximize the SERS effects. Recent developments in the synthesis of anisotropic nanoparticles have led to shapes such as nanocubes, nanorods, and nanostars [17], types of particles which contain sharp points that concentrate the electromagnetic field, acting as intrinsic hot spots for SERS [18,19]. In this regard, gold nanostars (GNS) has become a very popular investigation topic [20,21,22]. Indeed, the elongated branches of GNS allow them to maximize the number of possible hot spots leading to high enhancement factors [23]. Thus, they are widely used to boost SERS effect and the search for new synthesis, so their careful design and improvements are hot investigation topic nowadays. In the last years we have reported the synthesis of GNS obtained by means of a neutral surfactant, Triton x-100, as shape directing and protective agent, and we have demonstrated the possibility to finely tune their branch length and LSPR features by controlling the synthetic conditions. More recently, we have used them as an effective substrate for the realization of SERS nanotags for determination of fibroblast activation protein in fibroblasts. Moreover, it has also been demonstrated that the addition of a silver shell to branched gold nano-objects can further enhance their SERS responses, and that the enhancement is strictly connected to the extent of the silver layer [24,25,26,27]. Inspired by these works, we decided to apply a silver coating layer to our Triton based GNS to increase their SERS response towards environmental pollutants. We evaluated the enhancing performance of the GNS at increasing the thickness of the silver shell in order to find the optimal configuration to obtain the strongest SERS enhancement. The experimental results were interpreted and corroborated by means of a full field electrodynamics modelling. The optimized GNS@Ag were subsequently grafted on glass slides to prepare SERS chips that were fully characterized also in terms of SERS signal reproducibility. These sensors were successfully used to detect norfloxacin and thiram in water, as representative of different classes of emerging pollutants. Finally, a thin layer of PDMS was spin coated on SERS chips, demonstrating their ability to detect toluene in water.

## 2. Materials and Methods

### 2.1. Chemicals

Tetrachloroauric acid (30% in HCl, 99.99%), 2-[4-(2,4,4-trimethylpentan-2-yl)phenoxy]ethanol, sodium borohydride (≥98%), ascorbic acid (≥99%), silver nitrate (≥99%), 7-mercapto-4-methylcoumarin (≥97%), (3-aminopropyl)triethoxysilane (APTES) (≥98%), norfloxacin (≥98%), tetramethylthiuram disulfide (≥97%), ethanol (≥99.8%), toluene (≥99.7%), hydrochloric acid (≥37%), nitric acid (≥65%), sulphuric acid (≥95%), hydrogen peroxide (30 wt%) and ammonium hydroxide solution (≈29%) were purchased from Sigma Aldrich.

Microscopy cover glass slides 21 mm × 26 mm have been obtained from DEL Chimica and PDMS (Sylgard 184) was purchased from Dow Chemical.

Water was deionized and double distilled (ddH_2_O).

All the glassware that came in contact with nanoparticles was always cleaned with aqua regia for 15 min, then washed three times with bi-distilled water for 3 min under sonication and dried in an oven for 1 h at 140 °C.

### 2.2. Instruments

UV-Vis-NIR: absorption spectra of colloidal suspensions were taken with a Varian Cary 6000i spectrophotometer in the 300–1800 nm range. Spectra of functionalized glasses were obtained in the same range of colloidal suspensions, placing the slides on the Varian Cary 6000i spectrophotometer equipped with a dedicated solid sample holder.

Transmission Electron Microscopy (TEM): images of GNS and GNS@Ag samples were collected on a Jeol JEM-1200 EX II instrument on, respectively, 1:100 and 1:200 diluted solutions, with a 10 µL sample dropped on nickel grids (300 mesh) coated with a Parlodion membrane. 

Dynamic Light Scattering measurements (DLS): Zeta potential measurements of GNS and GNS@Ag were performed with a Zetasizer Nano-ZS90 (source: polarized He-Ne laser, 30 mW output power, vertically polarized) on 1 mL of colloidal suspension. 

Contact angle: static contact angle determinations were made with a KSV CAM200 instrument, with the water sessile drop method. 

Scanning Electron Microscopy (SEM): SEM images were taken from a Tescan Mira XMU variable pressure Field Emission Scanning Electron Microscope–FEG SEM (Tescan USA Inc., Warrendale, PA, USA), located at the Arvedi Laboratory, CISRiC, Pavia. Slides were mounted onto aluminium stubs using double sided carbon adhesive tape and then were made electrically conductive by coating them with a thin layer of Pt/Pd (nm) while in vacuum. Images were obtained at 25 kV using In-Beam Secondary electron detector to have higher spatial resolution.

Raman and SERS: Raman and SERS measurements were performed at room temperature using a Labram Dilor spectrometer equipped with an Olympus microscope HS BX40. Laser beam was the He-Ne red light at 632.8 nm and the spectrometer is equipped with a motorized xy stage on which the investigated samples are positioned. We used two different objectives: for colloidal solutions a 10× magnification while for SERS chips a 50× one, leading to spot sizes of 100 µm^2^ and 4 µm^2^, respectively. Spectral resolution is about 1 cm^−1^ and the laser incident power was about 5 × 10^4^ W/cm^2^. A cooled CCD camera is used as detector. 

### 2.3. Synthesis of Gold Nanostars (GNSs)

GNS were prepared following the seed growth procedure previously described [28]. Seeds were prepared in a vial by adding 5 mL of Triton-X-100 aqueous solution (0.2 M) and 5 mL of HauCl_4_ aqueous solution (4.5 × 10^−4^ M). Then 600 µL of an iced-cooled aqueous solution of NaBH_4_ (0.01 M) were quickly added to the pale-yellow solution of AuCl_4_^−^ prepared in the previous step. The resulting brown-orange solution was stored in an ice bath and needed to be used within 3 h. 

The growth solution was prepared starting from 50 mL of a water solution of Triton-X-100 (0.2 M) and adding, under magnetic stirring, 2500 µL of AgNO_3_ in water (0.004 M), 50 mL of aqueous HauCl_4_ (4.5 × 10^−4^ M), 1700 µL of an aqueous solution of L-Ascorbic Acid (0.0788 M) and 120 µL of the seed solution previously prepared. After this last addition, the suspension turns from pink to purple and blue, finally giving a grey colloid. At this point, the mixing was stopped.

### 2.4. Silver Coating of Gold Nanostars (GNS@Ag)

Silver coating on the GNS was performed by adding, to 30 mL of GNS solution under magnetic stirring, a small volume, varied between 75 and 600 µL, of AgNO_3_ (0.1 M) and an equivalent volume of Ascorbic Acid (0.1 M) [24]. The reduction in silver by ascorbic acid was initiated by the addition of NH_4_OH (60 µL). After a few minutes, the colour of the solution began to darken, turning from grey to brown. 

Silver-coated GNS samples (GNS@Ag) were named after the volume of AgNO_3_ added.

### 2.5. SERS Testing of GNS and GNS@Ag Colloidal Suspensions

SERS response of GNS and GNS@Ag colloidal suspensions have been tested with Rhodamine 6G (R6G), a well-known Raman reporter, as probe molecule: after adding 500 µL of an aqueous solution of R6G (10^−5^ M) to 500 µL of GNS and GNS@Ag colloidal suspension, SERS signal was immediately collected. 

The main Raman modes of R6G are peaked at 613, 775, 1184, 1312, 1364, 1512 and 1651 cm^−1^, corresponding to the C-H, C-O-C and C=C vibrations of aromatic rings [29]. 

### 2.6. Preparation of SERS-Chips

Functionalization of slides with APTES: the samples were prepared according to a reported method [30]. Glass substrates were cleaned for 30 min in freshly prepared Piranha solution (3:1 *v*/*v* H_2_SO_4_: H_2_O_2_ (30%)), washed three times with double distilled water in a sonic bath and oven dried. The slides were then immersed for 5 min in a 10% (*v*/*v*) solution of APTES in ethanol at 60 °C, washed twice with ethanol and once with ddH_2_O in a sonic bath and blow-dried with nitrogen.

Functionalization of slides with a SAM of GNS@Ag: APTES-functionalized glass slides were fully immersed in a GNS@Ag colloidal suspension for 15 h, washed three times in ddH_2_O without sonication and, after this step, samples were carefully dried under N_2_ flux. 

The functionalization of glass slides with GNS@Ag occurs thanks to an electrostatic interaction between our nanoparticles, with a negatively charged surface (ζ-potential = −26 (2) mV) and positively charged terminal amino groups of APTES. The amino moieties of APTES are positively charged in our work condition with GNS@Ag colloidal suspension at pH 3: to this purpose, before the immersion of glass slides, the pH of GNS@Ag colloidal solution was reduced from 9.50 to about 3, by adding HNO_3_ 0.1 M.

In a typical preparation, 8 glass slides were prepared at the same time in an 8-place glass staining jar.

### 2.7. Detection of Pollutants in Water

A 40 µL drop of pollutant solution was deposited on the top of the GNS@Ag SERS-chip and spread by covering the functionalized glass with a blank and clean glass slide of the same dimension, in order to obtain an almost homogeneous film of pollutant solution between the two glass slides. The so-assembled sample was quickly used for Raman analysis. 

Norfloxacin stock at 10^−3^ M was prepared in water and subsequently diluted to 10^−4^ M, 10^−5^ M, 10^−6^ M, 10^−7^ M and 10^−8^ M.

The main Raman modes of norfloxacin are peaked at 740, 1390 and 1620 cm^−1^, corresponding, respectively, to the vibration of C-F bond, the stretching of carboxylate and the stretching of C=C/C=N [31].

Thiram stock at 3.12 × 10^−5^ M (7.5 ppm) was prepared in water and subsequently diluted to 1 ppm, 0.50 ppm, 0.25 ppm, 0.10 ppm and 0.01 ppm. 

The main Thiram modes are peaked at 550 and 1350 cm^−1^ corresponding, respectively, to the stretching of C=S bond and the rocking of CH_3_ [32].

### 2.8. Detection of Thiram in Ethanol

Thiram stock at 3.12 × 10^−5^ M (7.5 ppm) was prepared in ethanol and diluted to 1 ppm, 0.50 ppm, 0.10 ppm and 0.01 ppm. 

SERS chips of GNS@Ag were immersed in ethanolic solution of Thiram for 1 h, washed copiously with ethanol for three times, without sonication, and dried under N_2_ flux [15].

### 2.9. Coating of GNS@Ag Monolayers with PDMS

A silicone elastomer base and curing agent were mixed together in the ratio of 10:1 (in wt%) and were kept shaking for 1 h. The PDMS elastomer was then diluted in tetrahydrofuran (THF) solution to obtain a concentration range from 1% to 10%. The thickness of the PDMS layer was controllable by changing the concentration of the PDMS elastomer. An amount of 100 µL PDMS elastomer solution was dropped onto the GNS@Ag glass slide, and the spin coating procedure was accomplished after 5 min at a spin speed of 1000 rpm with an acceleration of 500 rpm/sec. The coated films on the GNS@Ag monolayers were then cured in an oven at 30 °C for 15 h. The deposition was performed using a Spin 150 spin coater (SPS Europe).

### 2.10. Detection of Toluene

Firstly, a 5 mM solution of Toluene in water was prepared. Subsequently, a 20 µL drop of this solution was placed onto the SERS chip with PDMS and a SERS signal was immediately collected. 

Two main Toluene modes are peaked at 786 and 1031 cm^−1^ corresponding, respectively, to the bending and stretching of C-C bond. A third mode, due to the stretching of Ph-CH_3_, is located at 1210 cm^−1^.

## 3. Results and Discussion

### 3.1. Preparation and Optimization of GNS@Ag

#### 3.1.1. Preparation of GNS@Ag

GNS were prepared by a seed-growth method in which TRITON X100 acts both as shape-directing and as stabilizing agent using a well-established method we described previously in our papers [28]. Representative TEM images of GNS obtained in a typical preparation are shown in Figure S1, and their Uv-Vis-NiR spectrum is reported in Figure S2. As we have previously reported, the position of LSPR features can be tuned by changing the synthetic parameters: in this case we set the conditions to obtain a first resonance close to 900 nm and the second one close to 1500 nm, which was identified as the fundamental resonant mode. The resonance close to 900 nm corresponds instead to a higher order mode [33]. TEM images (see Figure S1) clearly show the presence of quite regular six- and five-branched objects together with less regular individuals, having a maximum tip-to-tip distance in the range between 60 and 90 nm. 

**Figure 1:**
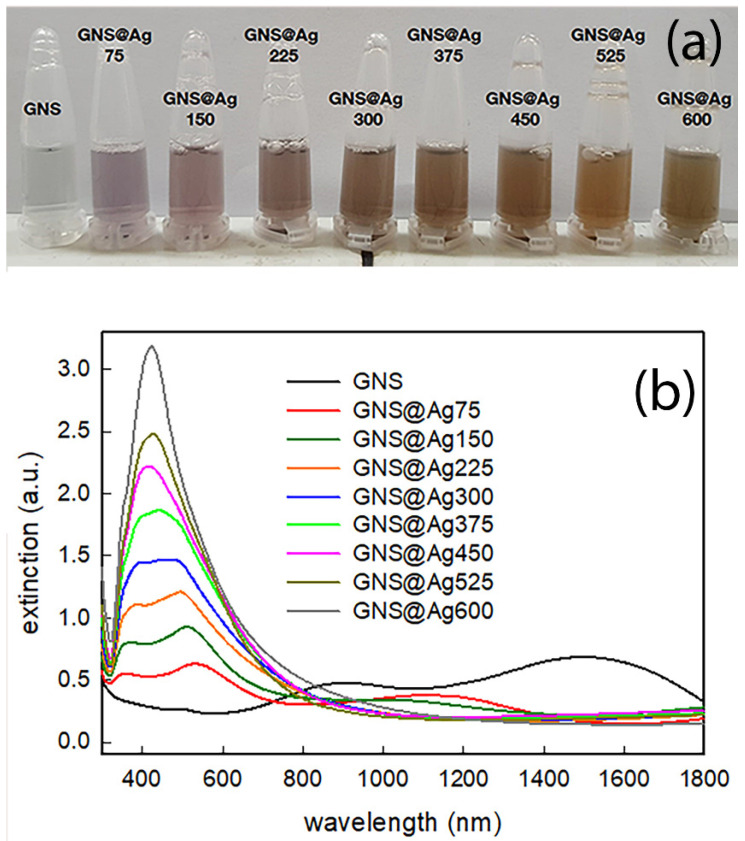
(**a**) photograph of colloidal suspensions (after dilution 1:3 with bidistilled water) of GNS (first vial) and GNS@Ag obtained with increasing silver/ascorbic acid addition; (**b**) UV-vis-NIR spectra of all the investigated samples.

Silver coating of these GNS was obtained by adapting the method proposed by Vo-Dinh and colleagues [24]. Briefly, silver nitrate, ascorbic acid and ammonia were added in variable amounts to as-obtained GNS. Success of coating with silver can be easily perceived by eye, with the dark blue suspension quickly turning to a reddish-brown colour, with different nuance and intensity as a function of the quantity of silver added, as can be seen from the photograph shown in Figure 1a.

UV-vis NIR spectra were taken for all the samples, together with TEM images.

As can be clearly seen by Figure 1b, addition of Ag causes remarkable changes in LSPR features. The LSPR band close to 1500 nm typical of GNS (see black line in Figure 1b) initially blue shifts (red line) for the smaller Ag quantity, and then it completely disappears. At the same time a new LSPR feature arises in the range between 400 and 550 nm, which blueshifts as silver and ascorbic acid content further increase. For higher additions, a LSPR feature close to 400 nm becomes predominant, suggesting that the main plasmonic resonances are due to nano-objects consisting mainly of silver.

**Figure 2:**
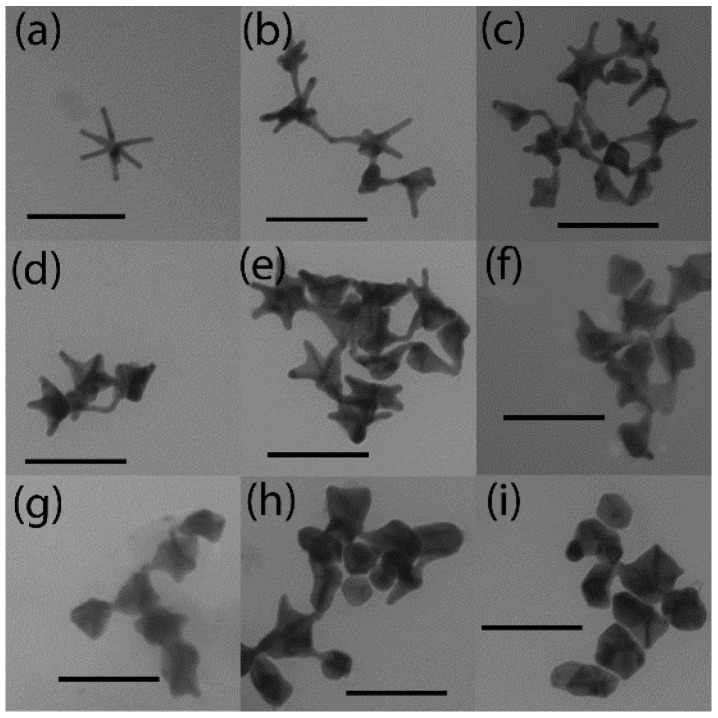
Representative TEM images of (**a**) GNS, (**b**) GNS@Ag75, (**c**) GNS@Ag150, (**d**) GNS@Ag225, (**e**) GNS@Ag300, (**f**) GNS@Ag375, (**g**) GNS@Ag450, (**h**) GNS@Ag525, (**i**) GNS@Ag600. Scale bar: 100 nm.

Analysis of TEM images in Figure 2 explains this spectral behaviour. Figure 2a reports the image of a typical GNS. For the lower values of silver/ascorbic acid concentrations, the coating mainly involves the GNS cores, leaving branches partially uncovered. As silver and ascorbic acid content increases, the GNS branches are progressively coated by silver. From GNS@Ag300 sample (Figure 2e) onward, branches of the majority of the original GNS appear almost completely covered, with a small portion of tips protruding from the silver envelops in a decreasing number of objects as the amount of silver increases. Indeed, for the higher investigated concentration of silver, sample GNS@Ag600, almost every object is completely enveloped by the silver shell and no tips are seen at all.

The SERS behaviour of the colloidal suspensions were investigated using Rhodamine 6G (R6G) as the Raman reporter. According to published data [34], R6G solutions exhibit intense Raman modes peaked at around 614, 766, 1178, 1306, 1361, 1509, and 1647 cm^−1^. The first is due to the in-plane bending vibrations of the aromatic ring. At increasing energy, we found features from CH, COC, and CC vibrations of aromatic rings.

**Figure 3:**
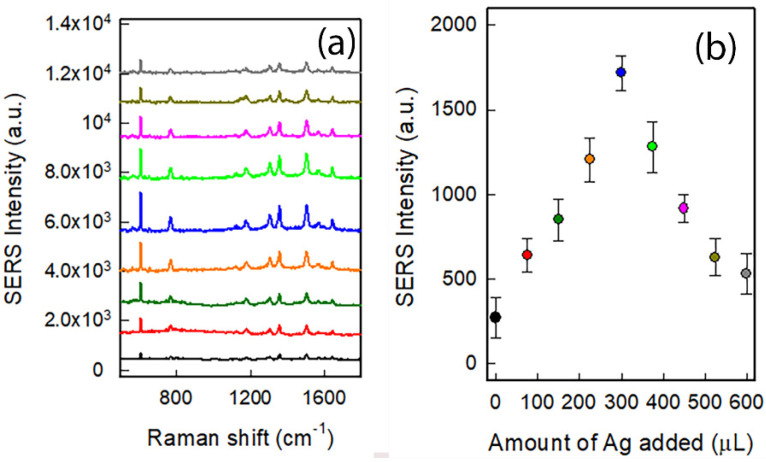
(**a**) SERS spectra of 10–5 M solution of R6G obtained in presence of colloidal suspensions of GNS (black line), GNS@Ag75 (red), GNS@Ag150 (dark green), GNS@Ag225 (orange) GNS@Ag300 (blue), GNS@Ag375 (green), GNS@Ag450 (pink), GNS@Ag525 (dark yellow), GNS@Ag600 (grey); (**b**) SERS intensity of mode at 610 cm^−1^ of R6G as a function of silver amount added to form the coating layer.

As can be observed from Figure 3a, the intensity of R6G SERS spectra increases with the amount of silver and subsequent coating layer extent, reaching a maximum for sample GNS@Ag300 (blue line) and then starting to decrease. It is important to stress that the concentration of nano-objects is the same in all samples, namely the one of starting GNS suspension. A 7-fold increase in the signal can be calculated by comparing the spectra collected for GNS@Ag300 with the one obtained with pristine GNS (black line): Figure 3b reports the intensities of the R6G peak at 610 cm^−1^. Comparing these results with the TEM data, one can observe that the higher SERS response is obtained for the configuration in which small parts of the original GNS branches protrude from the silver coating, i.e., the GNS@Ag300 sample, confirming the hypothesis of Vo-Dinh. On the contrary, the SERS signals weaken for higher silver quantities resulting in thicker silver coating layers progressively covering the protruding tips. In particular, the lowest values are observed when all the GNS tips are completely enveloped by silver in sample GNS@Ag600 (grey line).

#### 3.1.2. Theoretical Modelling

To fully understand this behaviour, we employed the boundary element method to model the experimental system as a six-branched gold nanostar surrounded by a silver shell of increasing thickness and immersed in water [35]. In our model, the silver spherical shell partially propagates along the arms of the gold star to better match the geometrical and compositional configuration emerging from the TEM images of Figure 2. The GNS geometry is characterized by an R_core_ = 10 nm core radius, an R_star_ = 65 nm external radius, a h = 8 nm thickness and a R_tip_ = 5 nm fingertip radius. The silver shell radius R_shell_ ranges from 20 nm to 70 nm, with a silver protrusion of length L_tip_≃15 nm when present. In all the studied cases, the material’s optical properties are taken from the literature [36,37,38], and the GNS@Ag target is illuminated with a plane wave normal to the nanostar equatorial plane and linearly polarized along the nanostar main axis. 

Figure S3 shows the extinction spectra for seven different GNS@Ag configurations, including a pure GNS and a GNS fully encapsulated in silver. The pristine GNS displays a strong main dipolar resonance at λ = 1645 nm, along with much weaker second and third order resonances at λ = 775 nm and λ = 620 nm. Additionally, the gold core resonance is also visible at λ = 520 nm. These findings are in excellent agreement with previously reported results [39], and nicely match the experimental spectrum of Figure 1, considering the spectral broadening due to size and shape dispersion. The addition of the silver shell leads to a dramatic blue-shift of the main dipolar resonance towards the λ = 600–1000 nm range, consistently with the trends of Figure 1, and to the appearance of the dipolar and quadrupolar resonance of the spherical silver shell around λ = 400 nm. Likewise, the GNS@Ag higher order resonances are pushed towards the high energy end of the spectrum and contribute to the creation of the double peak feature clearly visible in the silver rich samples of Figure 1.

**Figure 4:**
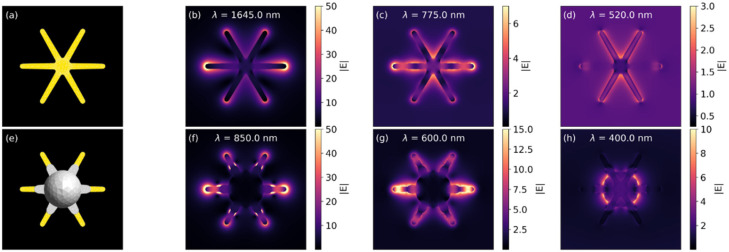
Local field enhancement maps for a pristine GNS and a R_shell_ = 30 nm GNS@Ag. The field is mapped over a square of side 200 nm at the GNS equatorial plane: (**a**,**e**) Sketch of the modelled structures; (**b**,**f**) local field map of the main dipolar resonance at λ = 1645 nm and λ = 850 nm; (**c**,**g**) local field map of the second order resonance at λ = 775 nm and λ = 600 nm; (**d**,**h**) local field map of the gold core and silver shell resonance at λ = 520 nm and λ = 400 nm.

We may better understand the nature of the observed plasmonic resonances by examining the corresponding local field maps displayed in Figure 4. Focusing first on the pure GNS, Figure 4b immediately confirms the dipolar nature of the λ = 1645 nm feature, with strong field hot spots located at the star tips. The higher order nature of the λ = 775 nm peak is instead highlighted in Figure 4c, where additional field maxima are present along the GNS main axis. Finally, the much weaker core resonance is barely detectable in Figure 4d. We can draw similar conclusions for GNS@Ag by looking at the field enhancement maps of the R_shell_ = 30 nm structure as a representative example. Moreover, in this case we notice from Figure 4f that the main λ = 850 nm resonance is dipolar in nature, but now the dipolar hot spots are confined to the protruding portion of the gold nanostar arm. Similarly, the field enhancement map associated with the higher order peak at λ = 600 nm displays an additional central maximum and is confined to a single GNS@Ag arm, with the novelty that the field enhancement now spills over the silver covered portion of the star branch (Figure 4g). Additionally, in contrast with the pure GNS case, the silver spherical enclosure can instead support a strong dipolar resonance at λ = 400 nm, closely resembling that of a single, isolated silver sphere (Figure 4h).

**Figure 5:**
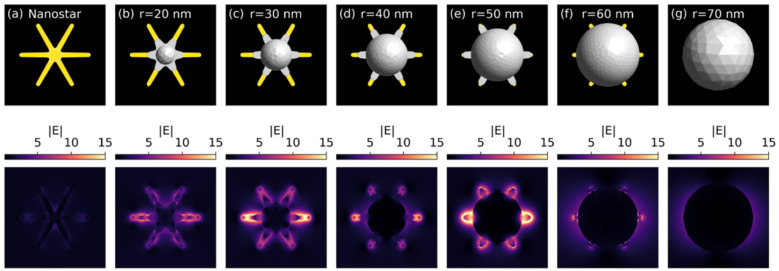
Local field enhancement maps for all the modelled structures at λ = 608 nm. (**a**): naked GNS; (**b**–**f**): GNS with increasing Ag shell thickness; (**g**) GNS completely enclosed by Ag shell of 70 nm thickness. The field is mapped over a square of side 200 nm at the nanostar equatorial plane.

Finally, we try to provide a robust interpretation for the SERS results observed in Figure 3b. To do so we map the field enhancements at λ = 608 nm for all the modelled structures. The results displayed in Figure 5 indicate that larger field enhancements are obtained as the main GNS@Ag resonance shifts toward the SERS excitation wavelength at λ~608 nm, and that the enhancement decays as soon as the nanostar branches are enclosed by the silver shell. A notable exception to this trend is seen in Figure 5c, where a higher order resonance perfectly matches the SERS excitation wavelength, nevertheless such an occurrence is likely to be averaged out in a realistic experimental scenario, where only the main spectral features of the GNS@Ag contribute to the visible trends.

To obtain a rough estimate of the SERS enhancement provided by each configuration, we compute the average of the fourth power of the field enhancement (|E|^4^) over each panel of Figure 5. The enhancement estimations reported in Figure S4 indicate that, with the already discussed exception of the R_shell_ = 30 nm case, the best performing structures are those whose main resonance is not quenched by the silver encapsulation and tuned towards the λ~608 nm SERS excitation wavelength. Conversely, an excess of silver leads to completely covered GNS, and to the ultimate suppression of their resonances.

### 3.2. Preparation and Characterization of GNS@Ag Based SERS Chips

Having confirmed and rationalized the excellent SERS responses of GNS@Ag300, we proceeded in the preparation of SERS chips. Reproducibility of the colloidal suspensions synthesis was checked, and it is reported in Figure S5. The first step of the chips preparation process was the formation of a monolayer of aminoproyltrietoxysilane (APTES) on microscopy cover glass slides by means of a reported procedure, with a simple immersion of the slides in a 10% APTES solution in methanol [30]. Success of silanization is evidenced by contact angle (c.a.) measurements, with an increase to a value of about 55 (5)° starting from a value <10° for the piranha cleaned starting glass slides.

From DLS measurements we found that colloidal suspensions of GNS@Ag300 show a z–potential of about −10 mV in the pH range between 4 and 8 (see Supplementary Material, Figure S6), and colloidal samples of GNS@Ag 300 can easily be brought to pH close to 4 by simply adding a stock solution of nitric acid. At this point, immersion of APTES functionalized glass slides into the colloidal suspension of GNS@Ag300 brought to pH 4 results in the protonation of pending –NH_2_ groups, allowing negatively charged nano-objects to electrostatically interact and to give a self-assembled monolayer (SAM) on the slides, yielding the glass-supported SERS chips. Formation of the SAM can be easily observed as the glass slides turn of a dark red-brownish colour. The obtained samples were characterized by means of SEM, UV-vis spectroscopy and SERS. 

**Figure 6:**
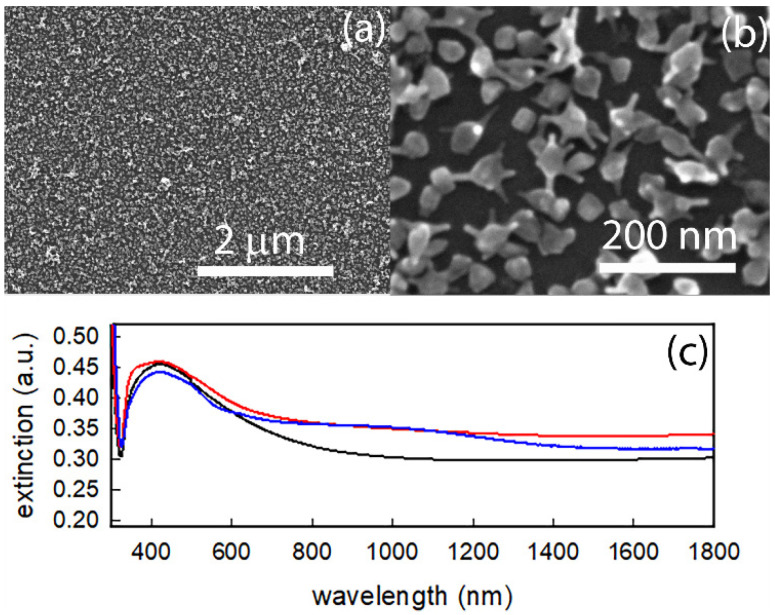
(**a**,**b**) SEM images of a GNS@Ag300 SERS chip; (**c**) spectra of three GNS@Ag300 SERS chips.

SEM images confirms the presence of a quite dense and homogeneous layer (Figure 6a) of GNS@Ag300 objects conserving their typical morphology (Figure 6b) observed with TEM images, with negligible presence of small visible aggregates. Uv-vis spectra of three samples coming from different preparation jars are reported in Figure 6c, showing a wide LSPR absorption in the overall investigated spectral region, probably caused by plasmonic coupling between objects, but dominated by a band in the range between 400 and 600 nm, almost superimposable to the features observed in the colloidal suspension’s spectra. Indeed, SEM and spectroscopic characterizations seem to indicate: (i) no changes in GNS@Ag upon grafting procedure; (ii) a good homogeneity of the GNA@Ag monolayer; (iii) good reproducibility of preparation; (iv) the presence of a dense packing which should lead to several hot spots between tips and/or cores owing to different adjacent objects. The quality of a SERS substrate is quantified by the so-called enhancement factor (EF), theoretically defined as the ratio between the SERS intensity and the Raman one [40]. In this regard, our samples exhibit in non-resonant conditions EF of the order of 10^7^ which is in good agreement with average EF values reported in literature [41]. For a detailed description of the calculus procedure, please refer to the Supporting Material.

To assess the homogeneity of the SERS response we used methylmercaptocumarine (MMC) as probe molecule due to its high Raman cross section and its ability to interact by means of strong covalent interactions with noble metal nano-objects, forming a SAM on the samples surface. Samples were functionalized with a 1 h immersion in an ethanol solution of MMC (10^−5^ M), washed three times with ethanol, without sonication, to remove any excess from the surface, and then dried under N_2_ flux before SERS measurement [15]. 

**Figure 7:**
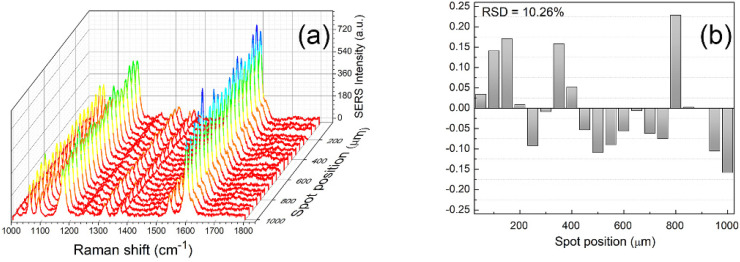
(**a**) SERS spectra obtained during a 1 mm-length linear scan of GNS@Ag300 chips coated with MMC; (**b**) relative RSD values obtained using the integrated intensities of mode at 1170 cm^−1^.

We performed multiple linear scans along the chips both on millimetre and micrometre scales, using a 50× objective and sampling lengths of the order of 1 mm and 30 µm, respectively. As reported in Figure 7 and Figure S7, the results clearly show a high homogeneity in the samples SERS response, obtaining RSD values of about 10% for both sampling scales. RSD values were obtained using the integrated intensities of the mode at 1170 cm^−1^ due to the symmetric benzene ring breathing. This band has been chosen because less affected by side bands and/or non uniform background signals [42]. Indeed, it is generally stated that RSD values below 15% indicate a good homogeneity of the SERS substrate [43].

### 3.3. Norfloxacin Detection

**Figure 8:**
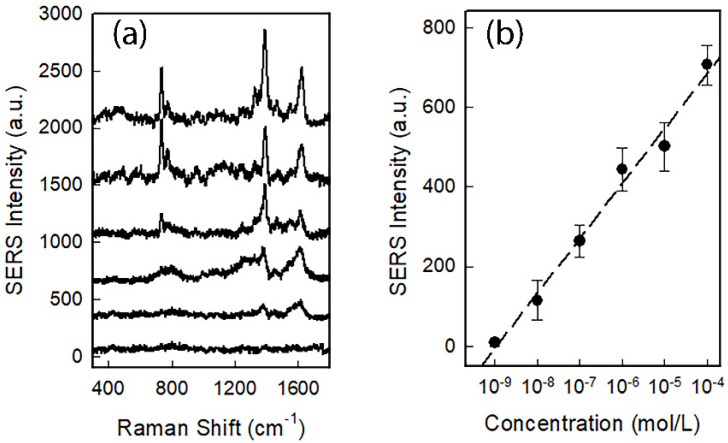
(**a**) SERS spectra of norfloxacin water solutions with concentration ranging from 10^−9^ (lower spectrum) to 10^−4^ M (upper spectrum) spotted on GNS@Ag300 SERS chips; (**b**) calibration curve obtained from mode at 1390 cm^−1^ (r^2^ = 0.987).

Having assessed the reliability of SERS chips, we tested them for norfloxacin detection. Norfloxacin solutions were prepared in water with concentration ranging between 10^−9^ and 10^−4^ M, and directly spotted on the SERS chips. The spectra were collected soon after the deposition, preventing the drying of the solution. Results are given in Figure 8a. One can clearly notice the presence of typical norfloxacin modes: the vibration of C–F bond at 740 cm^−1^, the stretching of carboxylate at 1390 cm^−1^ and the C=C/C=N stretching at 1620 cm^−1^ [7,8]. 

Figure 8b shows the calibration curve obtained with the SERS intensity of the mode at 1390 cm^−1^ as a function of the molar concentration. This plot clearly indicates that the SERS chips can detect norfloxacin in water, till the value of 10^−8^ M corresponding to 3 ppb. As the maximum residue limit set by EU is 3.01 × 10^−7^ M, corresponding to a value close to 100 ppb, we can affirm that the proposed SERS chips are optimal candidates for the rapid detection of dangerous concentrations of norfloxacin in water. 

### 3.4. Thiram Detection

Thiram sensing with the SERS chips was tested using two configurations, in order to face two different situations, i.e., thiram as a water pollutant and thiram as a residual pesticide on fruits. Thiram is, in fact, usually found on fruit peels. In this case, the sampling procedure consists of washing the fruits with ethanol [15]. Thus, we opted for a measurement setup in which SERS chips were immersed in ethanol solutions of thiram for a certain time. After incubation, thiram is expected to react with the silver surfaces to form a strong S–Ag covalent bond. Before collecting the Raman spectra, the incubated chips were properly washed and dried in order to eliminate any trace of solvent and unbound analytes, Raman spectra were taken. 

**Figure 9:**
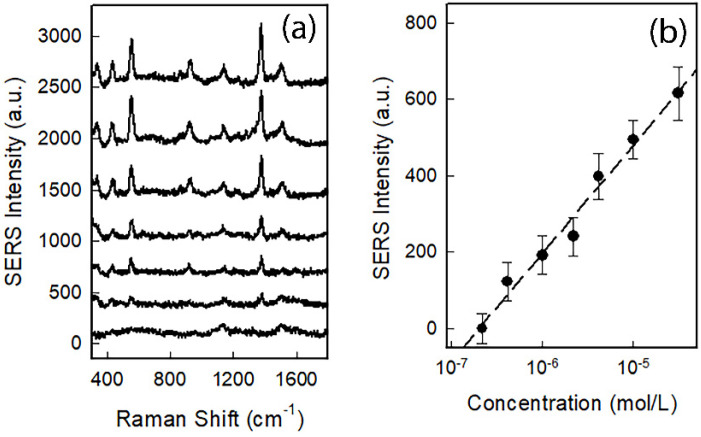
(**a**) Spectra of SERS chips after incubation in EtOH solutions of thiram with concentration ranging from 2.2 × 10^−7^ (lower spectrum) to 3.2 × 10^−5^ M (upper spectrum); (**b**) calibration curve obtained from mode at 1375 cm^−1^ (r^2^ = 0.982).

Thiram typical SERS spectra were observed, with two main modes at 550 and 1375 cm^−1^, as can be seen in Figure 9a. The pesticide is easily detectable for concentrations below 10^−5^ M following the SERS intensity of the mode at 1375 cm^−1^, with a limit of detection (LOD) which was estimated close to 0.4 µM, as can be seen in Figure 9b.

As regard the thiram detection in water samples, the same procedure performed for norfloxacin was used, recording the SERS signal soon after the drop deposition. Moreover, in this case thiram was easily detected in sub-ppm concentrations, with a LOD again estimated close to the value of 0.4 µM, corresponding to 100 ppb. The corresponding spectra are reported in Figure S8.

### 3.5. Toluene Detection

**Figure 10:**
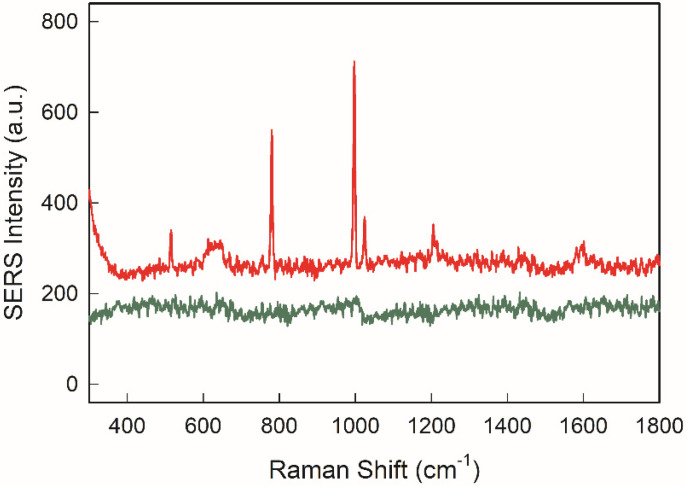
SERS spectra of a 5 mM solution of toluene in water spotted on 1% coated GNS@Ag SERS chip (red line), and on a GNS@Ag SERS chip (green line).

Toluene can be considered as representative of aromatic hydrocarbons which may have severe harmful effects on environment and health. Lacking the presence of any specific absorption group, these molecules interact awkwardly with the surfaces of noble metal nanoparticles, thus making their detection hard by means of SERS technique. The use of a PDMS film as a coating layer on the SERS active medium was introduced a few years ago. The addition of a water repellent polymeric film successfully allows the pre-concentration of aromatic hydrocarbons close to the SERS active surfaces thus enabling their subsequent detection. Inspired by this approach, we simply spin-coated a solution of PDMS base material diluted at 1% in THF on our SERS chips, achieving a PDMS thickness of about 100 nm. This thickness value is a good compromise between the efficiency of hydrocarbons trapping and the expected drop of the SERS effect due to the presence of the polymer acting as a spacer between the analyte and the SERS active layer. Higher concentrations (5 and 10%) of starting PDMS base material solutions were spun in the same conditions to verify the role of the layer thickness in toluene detection. SEM images after spin coating and curing steps show that the morphology of GNA@Ag monolayer was unchanged by the presence of the coating obtained with 1% PDMS solutions (see Figure S9), while coating with higher PDMS concentrations prevented to see the nano-objects under the thicker polymer layer. A reproducible value of contact angle over the whole surface of samples (101 ± 5°) accounted for homogeneous coating. Figure 10 reports the SERS spectrum collected on a drop of a 5 mM solution of toluene in water deposited on the 1% PDMS spin coated sample, together with the spectrum recorded on a pristine GNS@Ag uncoated SERS chip and on a blank glass.

As can be clearly observed, PDMS layer has a striking effect in revealing the presence of the typical Raman features of toluene, dominated by the presence of a mode at 1003 cm^−1^ which can be attributed to ring breathing, with other distinct features at 786 cm^−1^ (C-C bending), 1030 cm^−1^ (C-C stretching) and 1210 cm^−1^ (ring-CH_3_ stretching) [4]. Moreover, much weaker signals were recorded for sample obtained with higher thickness coming from more concentrated PDMS base material solutions (see Figure S10). This confirms that with the introduction of a pre-concentrating PDMS layer of proper thickness the GNS@Ag based SERS chips can be effective in detecting of aromatic hydrocarbons. Although absolutely preliminary, these measures further accounts for potentiality of our GNS@Ag SERS chips for a wide and flexible application to pollutants detection.

## 4. Conclusions

We performed a careful optimization of GNS@Ag synthesis to gain the maximum signal amplification compared to pristine GNS, with a robust explanation of experimental results obtained with boundary element method calculations. Having maximized the SERS signals obtainable, we prepared SERS chips based on chosen GNS@Ag and characterized their response in terms of homogeneity and reproducibility. Finally, we tested GNS@Ag based SERS chips to detect norfloxacin and Thiram, finding LOD values which can be considered more than satisfying. These results, even if preliminary, demonstrate how these sensing chips can act as a useful tool in the field of chemical sensing of pollutants for environment protection. The same goes for the PDMS-coated GNS@Ag substrates: our preliminary results confirm that these chips can be developed as a promising tool to detect targets which are non-specifically adsorbed onto metallic plasmonic nanostructures by profiting of preconcentration by a PDMS layer. 

## Data Availability

Not applicable.

## Supplementary Materials

The following supporting information can be downloaded at: https://www.mdpi.com/article/10.3390/nano12203609/s1, Figure S1: TEM images of GNS; Figure S2: UV-Vis-NiR spectrum of GNS; Figure S3: extinction spectra for all the modelled GNS@Ag structures; Figure S4: calculation of average of the fourth power of the field enhancement (|E|^4^) as a function of Ag shell thickness; Figure S5: Uv-Vis-NiR spectra of six GNS@Ag300 samples; Figure S6: z-potential values of GNS@Ag300 samples measured as a function of pH; Figure S7: reproducibility of SERS spectra obtained during a 1 mm-length linear scan of GNS@Ag300 chips; Figure S8: SERS spectra of thiram water solutions; Figure S9: SEM images of GNS@Ag chips coated with PDMS; Figure S10: SERS spectra of a 5 mM solution of toluene in water on SERS chips coated with different amounts of PDMS. Calculation of EF [22].
