# Supplementary material for: Surface-Enhanced Raman Spectroscopy Chips Based on Silver Coated Gold Nanostars
Source: Nanomaterials (Basel). 2022 Oct 14;12(20):3609. doi: 10.3390/nano12203609 (PMC9609606; doi:10.3390/nano12203609)
Supplement: Supplementary file 1 [file nanomaterials-12-03609-s001.zip › nanomaterials-1943604-supplementary.pdf]

Supplementary Material

Surface-Enhanced Raman Spectroscopy Chips Based on Silver Coated Gold Nanostars

Miriam Parmigiani [1], Benedetta Albini [2], Giovanni Pellegrini [2], Marco Genovesi [1], Lorenzo De Vita [1], Piersandro Pallavicini [1], Giacomo Dacarro [1], Pietro Galinetto [2] and Angelo Taglietti [1,*]

[1] Department of Chemistry, University of Pavia, Viale Taramelli 12, 27100 Pavia, Italy
[2] Department of Physics, University of Pavia, Via Bassi 6, 27100 Pavia, Italy
* Correspondence: angelo.taglietti@unipv.it

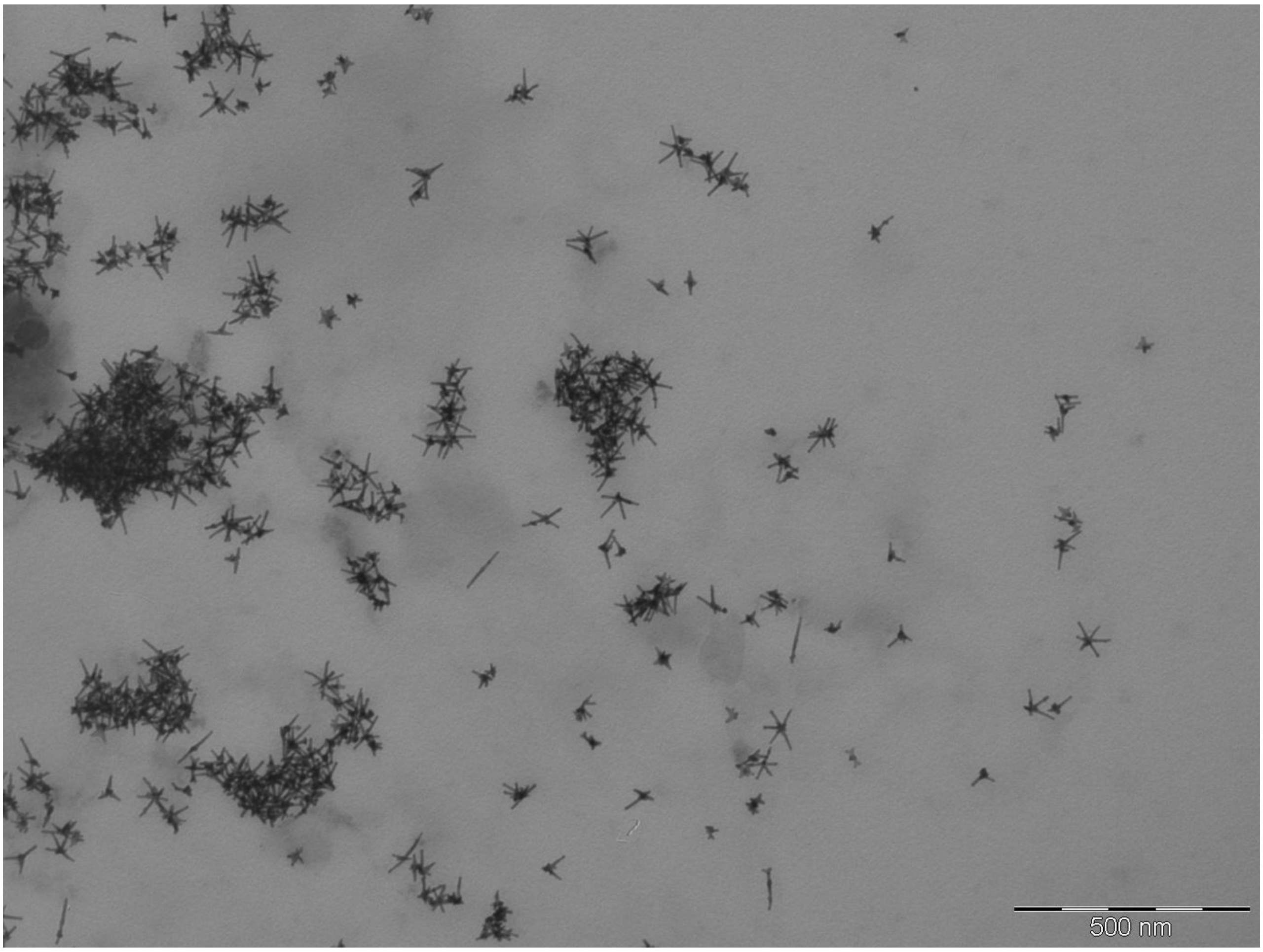

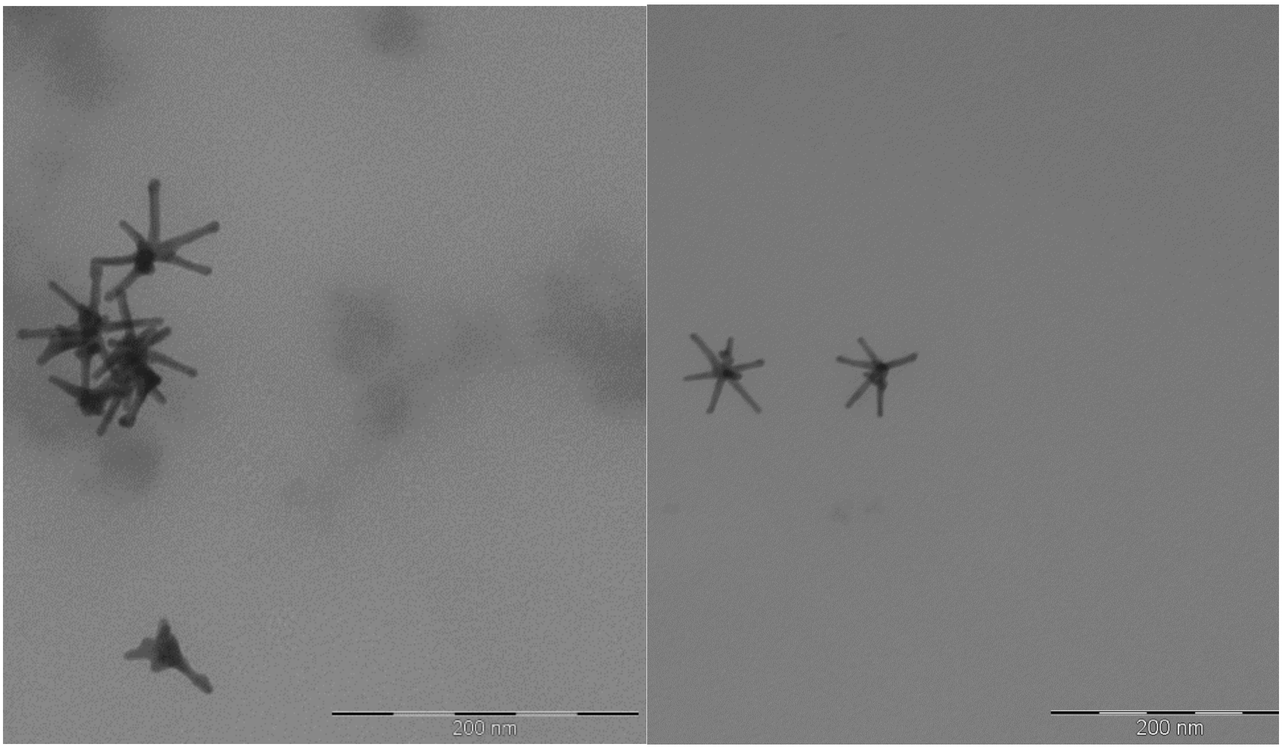

Figure S1. Representative TEM images of a GNS colloidal suspension used in this work.

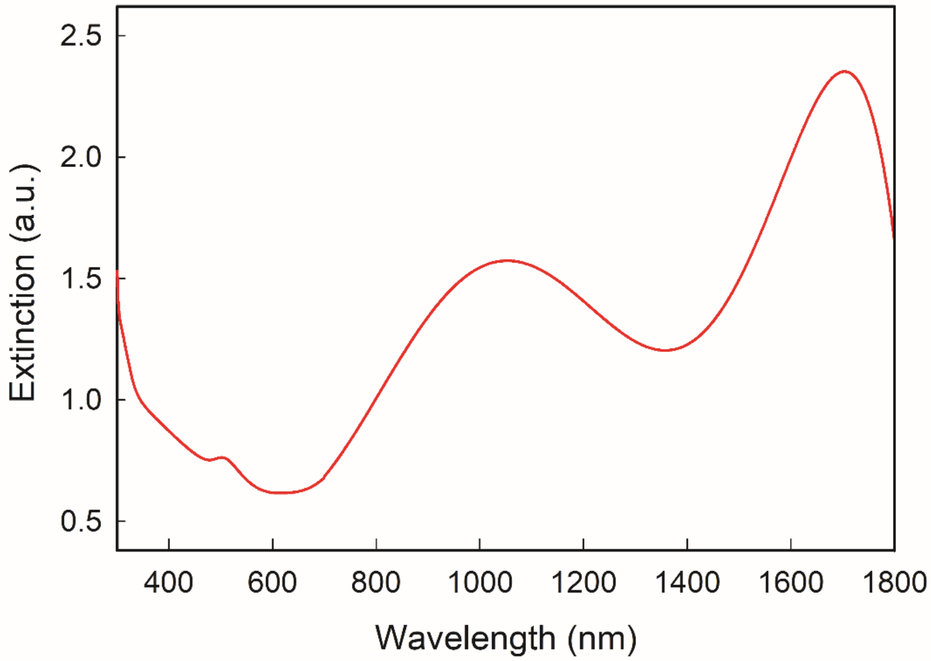

Figure S2. UV–Vis–NiR spectrum of a GNS colloidal suspension used in this work.

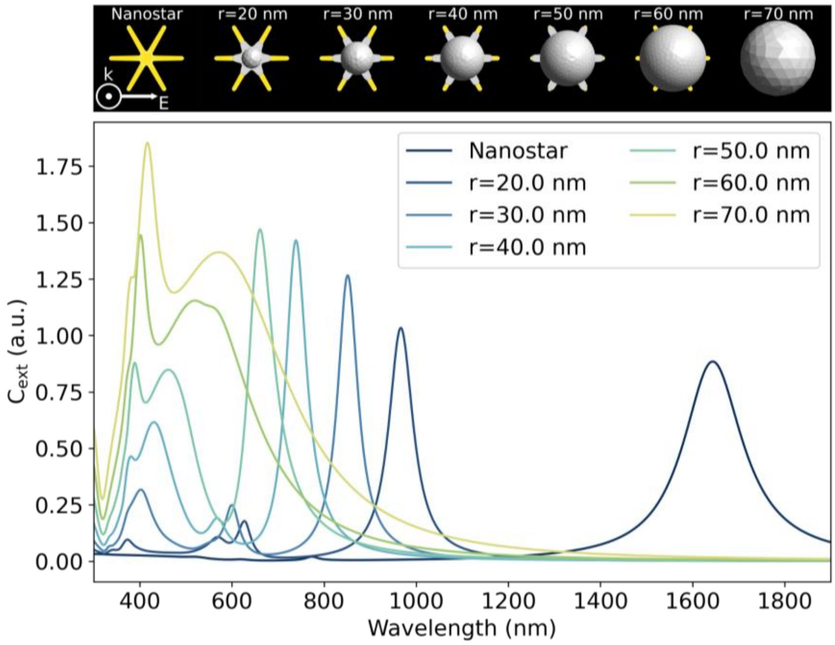

Figure S3. Extinction spectra for all the modelled GNS@Ag structures. The top inset displays each of the considered geometries, along with the configuration of the illumination field. .

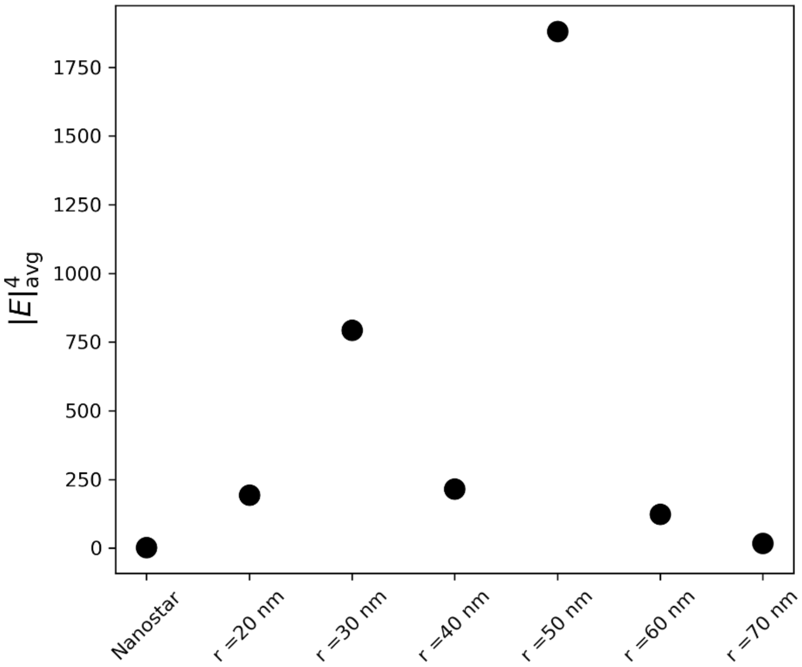

Figure S4. calculation of average of the fourth power of the field enhancement (|E|⁴) as a function of Ag shell thickness according to local field enhancement maps for all the modelled structures reported in figure 5 (see main).

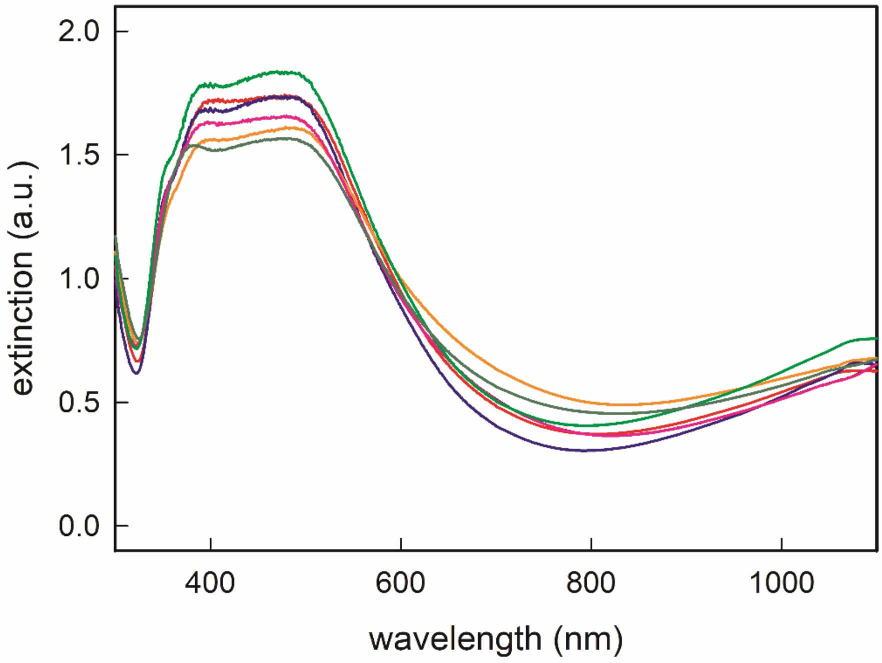

Figure S5. Uv–Vis–NiR spectra of GNS@Ag300 samples coming from six different preparations.

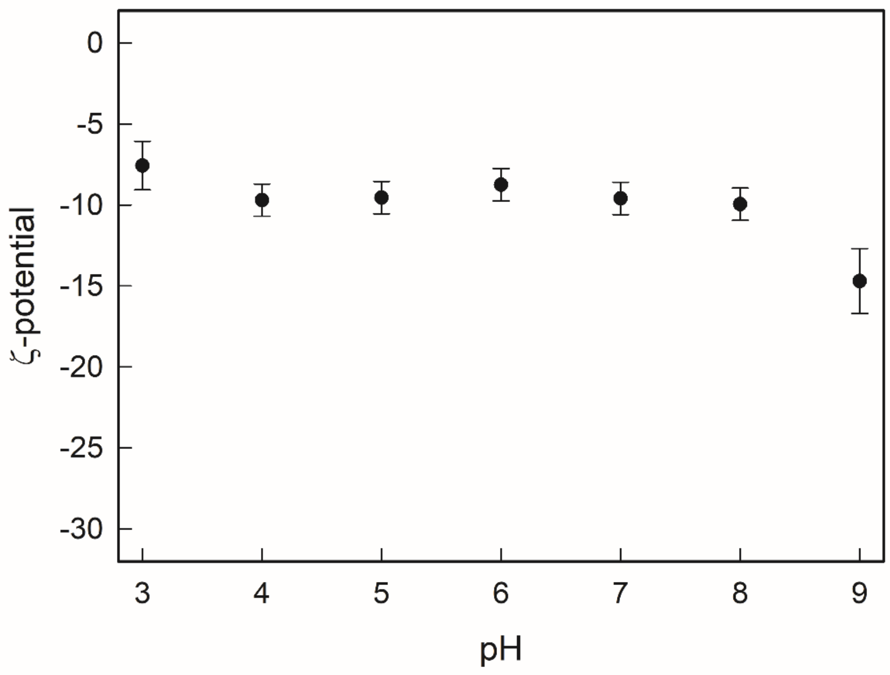

Figure S6. z-potential values of GNS@Ag300 samples measured as a function of pH.

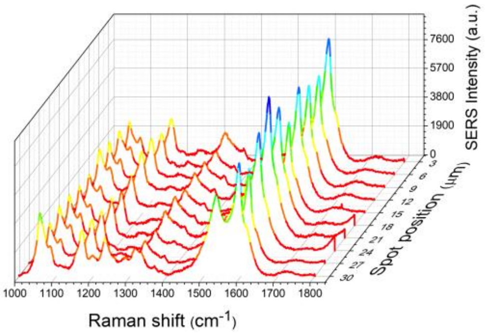

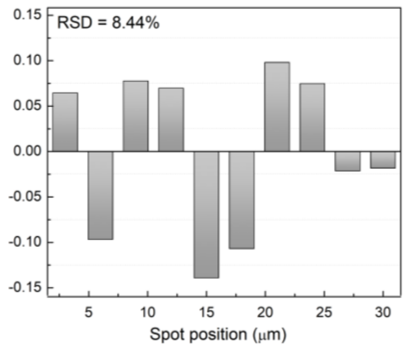

Figure S7. left: SERS spectra obtained during a 1 mm-length linear scan of GNS@Ag300 chips coated with MMC; right: relative RSD values obtained using the integrated intensities of the mode at 1,170 $cm^{-1.}$

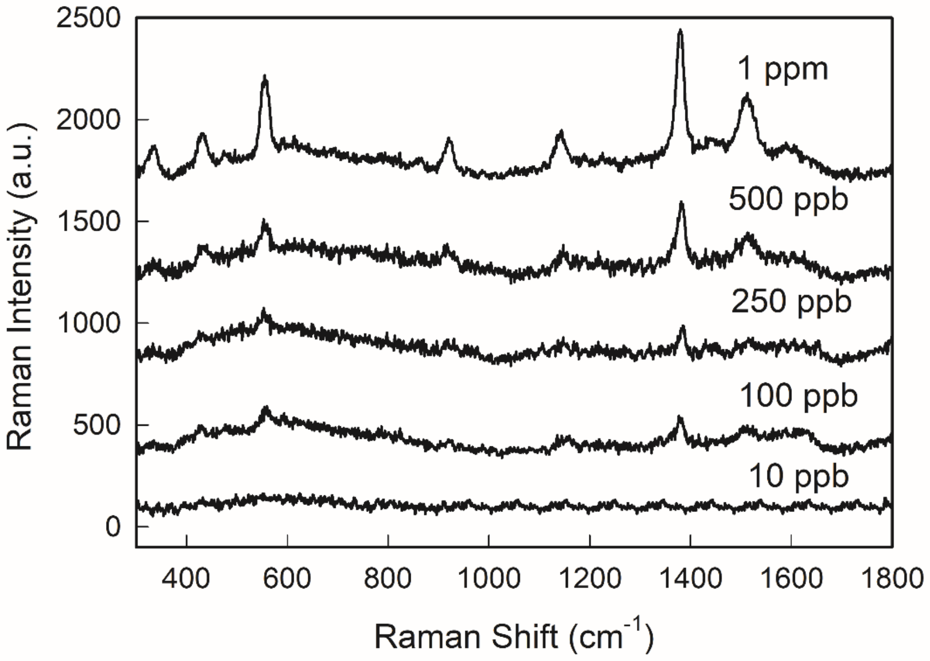

Figure S8. SERS spectra of thiram water solutions with concentration ranging from 1 ppm (upper spectrum) to 10 ppb (lower spectrum) spotted on GNS@Ag300 SERS chips

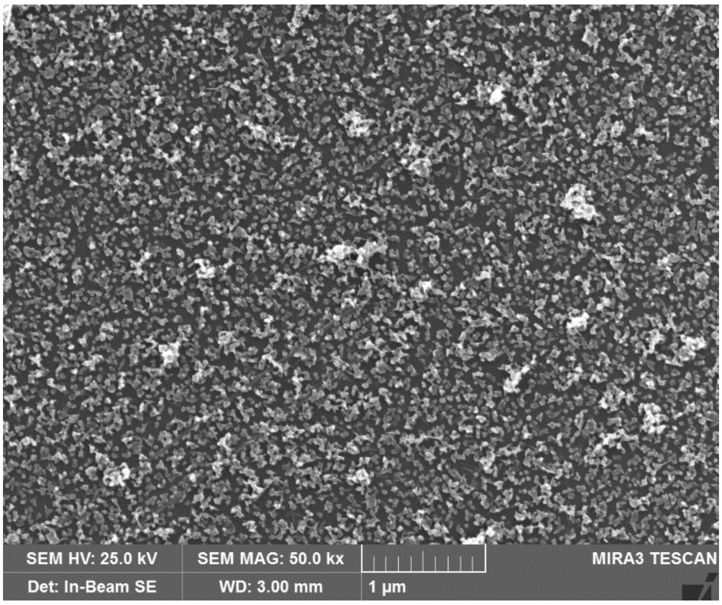

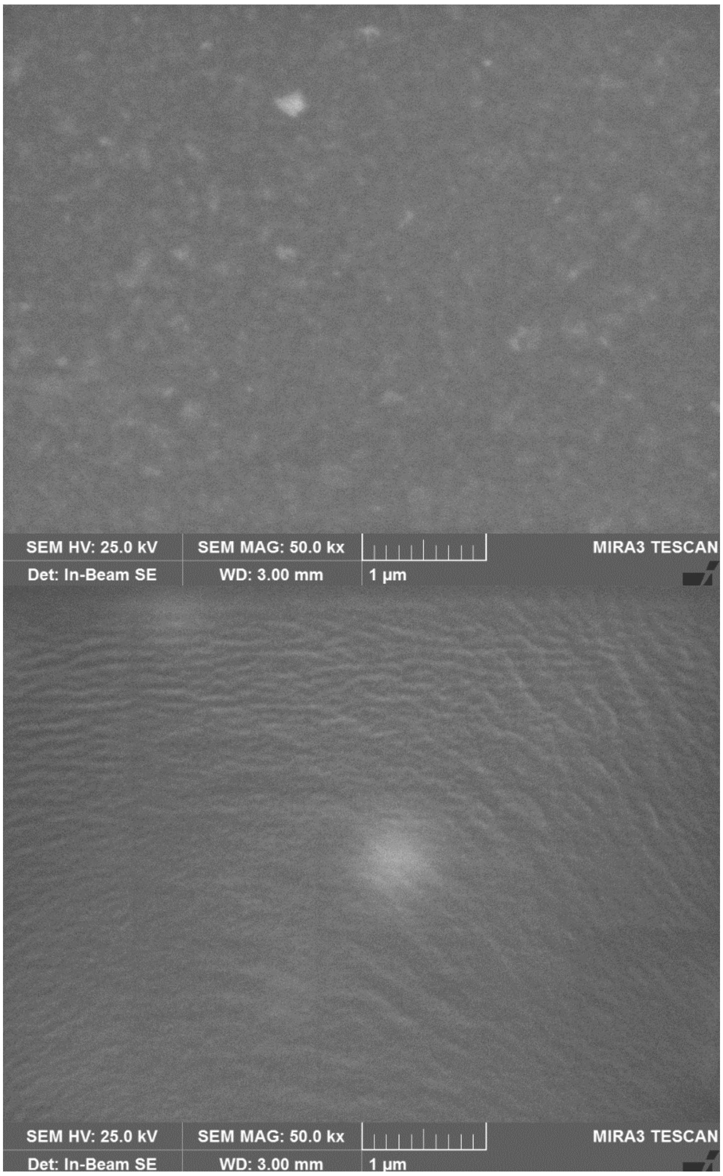

Figure S9. SEM images of GNS@Ag chips coated with PDMS using: 1% PDMS solution (up); 5% PDMS solution (middle); 10% PDMS solution (bottom).

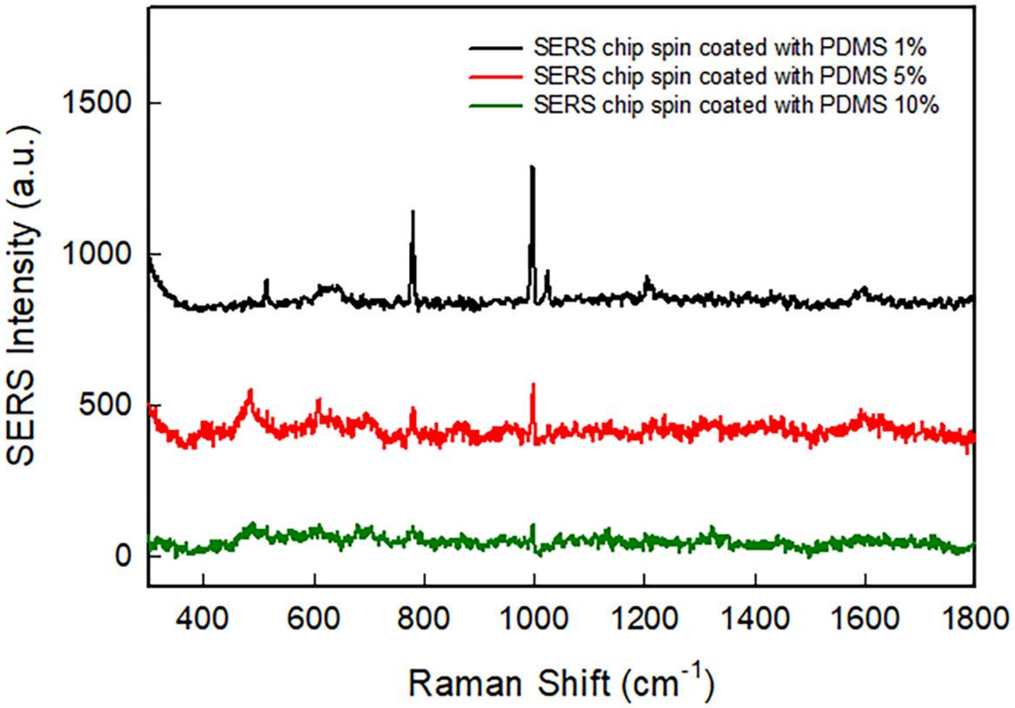

Figure S10. SERS spectra of a 5 mM solution of toluene in water spotted on 1% coated GNS@Ag SERS chip (black line), 5% coated GNS@Ag SERS chip (red line), 10% coated GNS@Ag SERS chip (green line).

Calculation of Enhancement Factor

To evaluate the EF, we deposited an 80 μL drop of 10^{-5} M R6G solution on a SERS substrate and another 80 μL drop of 10^{-3} M R6G solution on a blank glass slide in order to collect the R6G SERS and Raman response, respectively. In both cases, the drops were spread by covering them with a blank and clean glass slide to create an almost homogeneous film between the two glass slides.

We operatively used the following formula for the EF estimation:

$$EF = \frac{I_{SERS}}{I_{Raman}} \frac{c_{Raman} H_{Raman}}{c_{SERS} H_{SERS}}$$

where I_{SERS} and I_{Raman} are the integrated intensities of the R6G mode that falls at 610 cm^{-1} and c_{SERS} and c_{Raman} the molar concentration of R6G solution in both SERS and Raman measures, respectively.

Since the geometry of the experiment is the same for both SERS and Raman measures, and thus the area of the scattering volume covered by the laser spot is the same, the only parameter that counts is the height of scattering volumes. For Raman measure, we previously estimated that this height (H_{Raman}) is about 18 μm, while for SERS one, we demonstrated [1] that the SERS signal practically disappears for any distance longer than 10 nm (H_{SERS}).

Reference

1. Bassi, B.; Albini, B.; D'Agostino, A.; Dacarro, G.; Pallavicini, P.; Galinetto, P.; Taglietti, A. Robust, Reproducible, Recyclable SERS Substrates: Monolayers of Gold Nanostars Grafted on Glass and Coated with a Thin Silica Layer. *Nanotechnology* **2019**, *30*. https://doi.org/10.1088/1361-6528/aae9b3.
